# Shape‐Adaptability and Redox‐Switching Properties of a Di‐Gold Metallotweezer

**DOI:** 10.1002/chem.202100794

**Published:** 2021-05-21

**Authors:** Susana Ibáñez, Eduardo Peris

**Affiliations:** ^1^ Institute of Advanced Materials (INAM), Centro de Innovación en Química Avanzada (ORFEO-CINQA). Universitat Jaume I. Av. Vicente Sos Baynat s/n Castellón 1271 Spain

**Keywords:** gold, host-guest chemistry, metallo-tweezers, redox-switchable binding, supramolecular

## Abstract

The use of a carbazolyl‐connected di‐gold(I) metallotweezer for the encapsulation of several electron‐poor organic substrates, and a planar Au(III) complex containing a CNC pincer ligand, is described. The binding affinity of the receptor depends on the electron‐deficient character of the planar guest, with larger association constants found for the more electron‐poor guests. The X‐ray diffraction molecular structures of two host:guest adducts show that the host approaches its arms in order to facilitate the optimum interaction with the surface of the planar guests, in a clear example of an guest‐induced fit conformational arrangement. The electrochemical studies of the encapsulation of N,N’‐dimethyl‐naphthalenetetracarboxy diimide (NTCDI) show that the redox active guest is released from the receptor upon one electron reduction, thus constituting an example of redox‐switchable binding.

## Graphical abstract

**A di‐gold metallo‐tweezer** is used as receptor of a series of electron‐deficient planar organic guests, including three organic molecules and two square‐planar metal complexes. The host adapts its shape to maximize the attractive interaction with the guests. Redox‐active guests are released upon one electron reduction, thus this tweezer constitutes an interesting example of redox‐switchable binding.

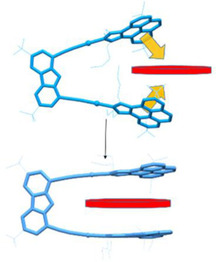

## Introduction

In the 1990s several research groups around the world reasoned that U‐shaped molecules with aromatic side walls would form rigid and highly preorganized concave cavities suitable for hosting aromatic guest molecules through π‐π stacking interactions.^[1]^ These supramolecular tools, which were called ‘molecular tweezers,^[1d]^ were predominantly organic‐based molecules that were efficient for the encapsulation of cationic or neutral guests with acceptor groups.^[1b]^ In addition, their unique U‐shaped structure endowed these supramolecular receptors with the ability to interact with biomolecules such as basic amino acids on proteins, therefore offering additional targets for medicinal chemistry.^[2]^ During the last two decades, starting with the pioneering studies by Bosnich and co‐workers in 2001,^[3]^ metal‐containing tweezers gained popularity due to their simplified modular synthesis relative to their organic congeners.[^3^, ^4^] In addition, the introduction of metals into the structure of the tweezer, endows new properties that can be used for a number of interesting applications. For example, the binding affinities of the luminescent alkynylplatinum (II) containing tweezers described by Yam[^4b^, ^4f^, ^5^] and Wang[^4h^, ^6^] were found to be perturbed by donor‐acceptor (D−A) charge transfer (CT) and metal‐metal and electrostatic interactions, producing dramatic color changes, with diverse applications that include, for example, the amplification of chiroptical signals^[6d]^ and visible‐light photocatalytic transformations.^[7]^

**Scheme 1:**
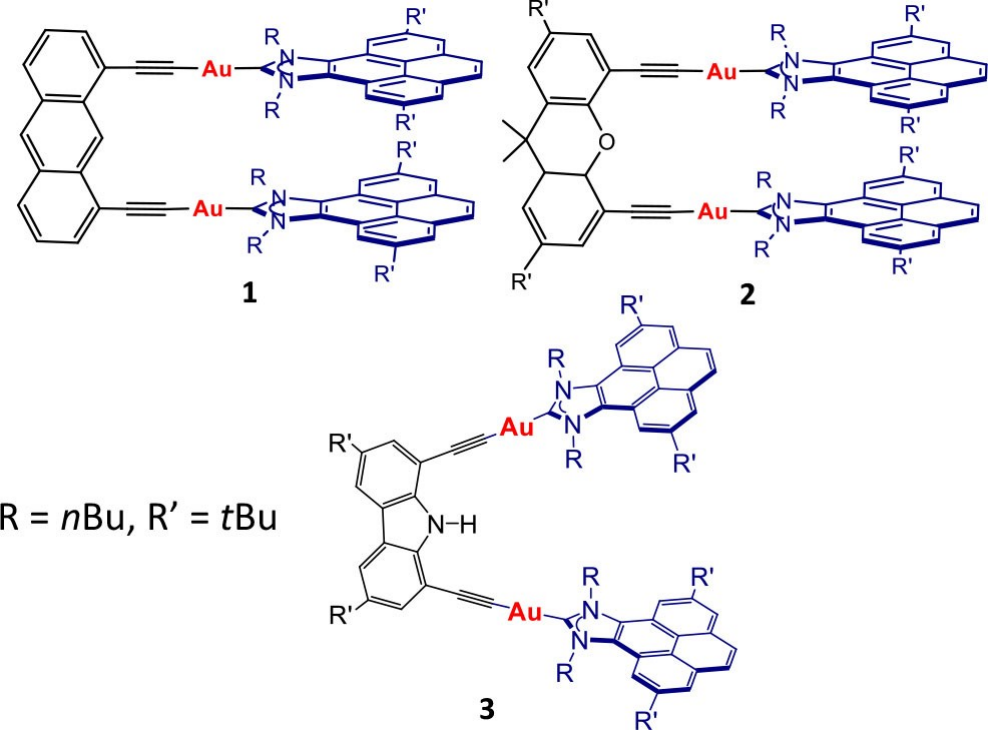
Three metallotweezers with pyrene‐imidazolylidene‐gold(I) arms.

During the last four years we contributed to the preparation of three families of metallotweezers, which contained arms constituted by gold(I)‐NHC moieties (NHC=N‐heterocyclic carbene decorated with polyaromatic systems), and three different bis‐alkynyl linkers (Scheme 1).^[8]^ In our studies, we observed that the supramolecular properties of these systems depended greatly on the nature of the linker used. In particular, the anthracenyl‐linked complex **1** showed a great tendency to self‐aggregate in non‐polar solvents and in the presence of cations such as Cu^+^, Ag^+^ and Tl^+^.^[9]^ The xanthenyl‐linked complex **2** showed interesting coordinating abilities that were highly dependent on the type of metal used.[^9b^, ^10^] Finally, the carbazolyl‐linked complex **3**, was able to encapsulate planar polycyclic aromatic hydrocarbons (PAHs), displaying larger associating constants for those planar guests functionalized with groups able to hydrogen bond with the NH group of the carbazolyl spacer.^[11]^ Noteworthy, all our host‐guest chemistry studies with **3** were performed using electron‐donor planar guests, thus the association constants that we found were rather low. Based on these previous results, we now decided to use the molecular tweezer **3** for the encapsulation of electron‐deficient planar aromatic guests. The encapsulation of such guest molecules should render discrete favorable donor‐acceptor‐donor (D‐A‐D) interactions with the potential to produce aromatic charge‐transfer interactions that can turn into interesting electronic and optical properties. In addition, herein we also describe the study the encapsulation of two planar Au(III) and Pt(II) complexes bearing pincer CNC ligands. Our motivation to perform such studies is because the encapsulation of planar *d* ^8^ and *d* ^10^ metal complexes in the cavity of a metallotweezer may produce interesting photophysical properties as a consequence of the appearance of non‐covalent metal‐metal interactions.[^5a^, ^5b^, ^12^]

## Results and discussion

**Scheme 2:**
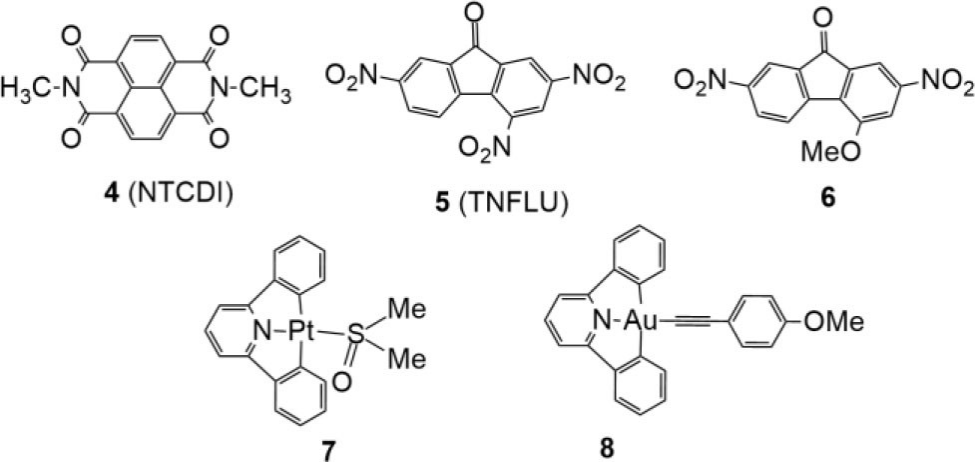
Guest compounds used in the study.

Scheme 2 shows the different compounds that were used as guests to form the corresponding inclusions complexes with **3**. We thought that the electron‐rich environment provided by the pyrene moieties in complex **3** could be used for encapsulating planar electron‐deficient organic molecules such as N,N′‐dimethyl‐naphthalenetetracarboxy diimide (NTCDI, **4**), 2,4,7‐trinitro‐9‐fluorenone (TNFLU, **5**) and 2,7‐dinitro‐4‐methoxy‐fluorenone (**6**). We deliberately obtained **6** from **5** (see experimental details in the Supporting Information file) to study if the replacement of one electron‐withdrawing nitro group by an electron‐donating methoxy group would have any measurable influence in the binding affinity of the guest. Together with these three organic guests, we also studied the binding affinities of the two square planar Pt(II) and Au(III) complexes **7** and **8**.

To quantify the binding affinities between **3** and compounds **4**–**8**, we performed ^1^H NMR titrations in CDCl_3_ at a constant concentration of **3** (0.5 mM). In general, the NMR titrations showed that the addition of the guests induced perturbations in several resonances of the protons of **3**. In particular, the signals due to the protons of the pyrene moiety and the resonance due to the methylene group bound to the nitrogen of the imidazolylidene of **3**, were shifted upfield upon addition of the different guests. Only in the case of the titrations with **5** and **6**, it was observed that the resonance of the proton bound to the nitrogen of the carbazolyl linker was shifted downfield by 0.9 and 0.2 ppm, respectively, suggesting that for these two guest molecules the formation of the host‐guest complex may be stabilized by the presence of hydrogen bonding interactions, most likely with the oxygen atoms from the nitro groups of the guests. In the case of the titration with the Pt(II) complex **7**, the titrations did not show changes in the spectrum of **3**, thus indicating that for this guest the formation of an inclusion complex was not detected. Based on the changes observed for the titrations with all other four guests, the association constants were determined by global fitting analysis.^[13]^ The data was best fitted to a 1 : 1 host:guest stoichiometry, as a result from the analysis of the distribution of the residuals compared to a 1 : 2 stoichiometry. The results indicate that the binding affinities of the guests are in the order **7**<**8**<**6**≪**4**<**5** as can be observed from the values shown in Table 1. The larger binding affinities found for TNFLU (**5**) and NTCDI (**4**) are in agreement with the stronger electron‐deficient character of these two planar molecules. Interestingly, the replacement of one of the nitro groups in **5** by a methoxy group results in a dramatic decrease of the association constant, as can be observed comparing the constants shown for **5** and **6** (compare entries 2 and 3 in Table 1). The Au(III) complex **8** shows a binding constant of 118 M^−1^, while the platinum (II) complex **7** did not show any measurable binding affinity with **3**. This result may be ascribed to the larger steric hindrance produced by the DMSO ligand bound to **7**, which makes the complex deviate from being fully planar. The solubility of both **3** and **8** allowed to perform a ^1^H NMR titration in toluene‐*d* ^8^, and the association constant that resulted from the nonlinear fitting of the corresponding binding isotherm was 687 M^−1^, therefore significantly larger than the one obtained in CDCl_3_. The results shown in Table 1 reflect that the metalotweezer **3** is an effective receptor of electron‐poor planar molecules, showing larger binding affinities than other molecular receptors for the same guests. For example, the constant found for the encapsulation of TNFLU is significantly larger than the association constants shown by other molecular tweezers with electron‐rich arms for the same guest under the same experimental conditions,^[14]^ and very similar to the one displaying the largest constant.^[15]^ With regard to the encapsulation of metal complexes, some authors previously showed how metallotweezers containing cationic alkynylplatinum arms displayed large association constants with several neutral and anionic guest planar metal complexes,[^3a^, ^5b^, ^6c^, ^7^] including **7** and **8**.^[5b]^ In particular, by using their alkynylplatinum‐containing tweezer, Yam and co‐workers observed that the association constant with **8** was found to be logK=4.43 under the same experimental conditions where we obtained an association constant of 118 M^−1^ for the encapsulation of the same metal complex with **3**.^[5b]^ The lower affinity found in our case is very likely due to the presence of the electronrich pyrene moieties, compared to the electron‐deficient planar cationic arms present in the Yam's case, which are more prone to favor the electrostatic interaction with planar electronrich metal complexes. In addition, in the Yam's case, the binding affinity between the host and the guest is strongly perturbed by Pt⋅⋅⋅M interactions,^[5b]^ whereas metallophillic interactions seem not to play a role in the formation of the host‐guest complex **8**@**3**.

**Table 1:** Association constants (M^−1^) for the complexation of **2** with guest.^[a]^

Entry	Guest	K_11_
1	**4** (NTCDI)	1014±30
2	**5** (TNFLU)	2069±105
3	**6**	170±3
4	**7**	–
5	**8**	118±2
6^[b]^	**8**	687±31

[a] K_11_ values calculated by global nonlinear regression analysis.^[13]^ Titrations carried out by ^1^H NMR spectroscopy, using constant concentrations of **2** (host) of 0.5 mM in CDCl_3_ or toluene‐*d* ^8^ [b] at 298 K. Errors refer to the non‐linear regression fittings.

**Figure 1:**
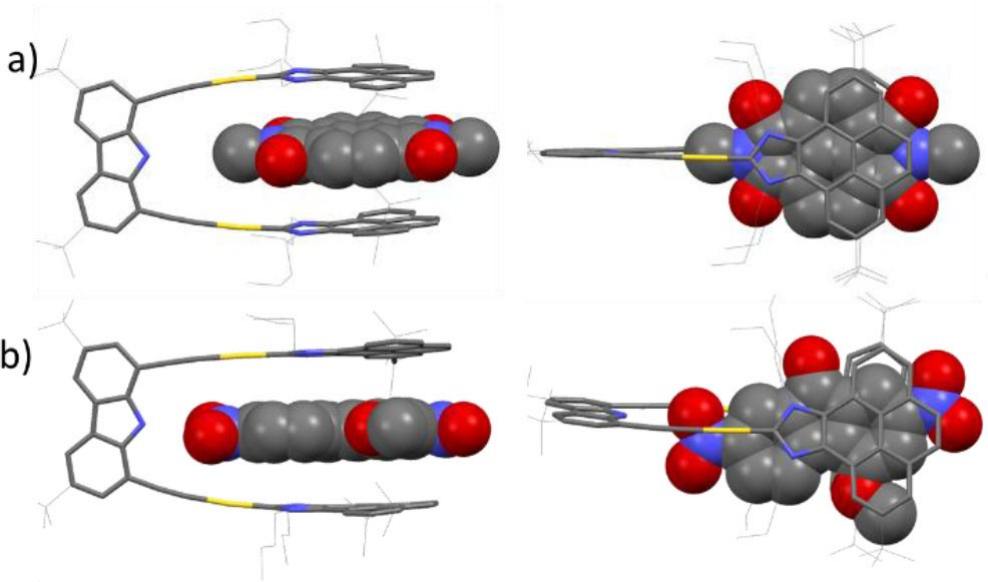
Two perspectives of the molecular structures of a) **4**@**3** and b) **6**@**3** obtained from single crystal X‐ray diffraction studies. Deposition numbers 2065669 (**4**@**3**) and 2065771 (**6**@**3**) contain the supplementary crystallographic data for this paper. These data are provided free of charge by the joint Cambridge Crystallographic Data Centre and Fachinformationszentrum Karlsruhe Access Structures service.

**Figure 2:**
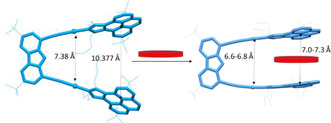
Induced‐fit conformational distortions displayed by **3** upon encapsulation of a planar electron‐deficient guest. The distances between the pyrene moieties are obtained from the separation between their centroids. The distances for empty **3** were taken from its molecular structure previously reported by us.^[11]^

Crystals suitable for X‐ray diffraction studies of **4**@**3** and **6**@**3** were obtained by slow diffusion of hexane into CHCl_3_ solutions containing equimolecular amounts of **3** and the electron‐deficient guest molecules. The molecular structures of these two host‐guest adducts (Figure 1) show that the planar guest molecule is sandwiched between the two pyrene arms of the metallotweezer. The distance between the two gold atoms in **4**@**3** is 6.813 Å, and the distance between the planes of the two pyrene cofacial units is 7.354 Å, as measured from the distance between the two centroids of the pyrene units. These distances are a little shorter in **6**@**3**, which shows an Au⋅⋅⋅Au separation of 6.645 Å and a distance of 6.956 Å between the two planes formed by the pyrene units. At this point it is important to mention that the molecular structure of ‘empty’ **3**, displays a Au⋅⋅⋅Au distance of 7.38 Å, and the distance between the two centroids of the pyrene units is 10.377 Å.^[11]^ The shorter distances shown by **4**@**3** and **6**@**3** reflect the great flexibility of the molecular host **3**, which is able to adapt its shape in order to maximize the face‐to‐face overlap with the electron‐deficient guest molecules, thus providing an interesting example of a guest‐induced conformational distortion of a host molecule (Figure 2).^[16]^

The molecular structure of **6**@**3** shows a short distance of 2.183 Å between the hydrogen bound to the nitrogen of the carbazolyl linker of **3** and one of the oxygen atoms of one of the nitro groups of the guest, therefore indicating the existence of a hydrogen bonding interaction between the host and the guest, as was predicted by the NMR titrations.

We wanted to have an estimation of the charge transfer produced in the interaction between the electron‐donating host and the different electron deficient guests upon host‐guest formation. Unfortunately, even the highest association constants that we found (in the range of 10^3^ M^−1^) were not large enough to produce significant amounts of the host‐guest complexes at the concentrations used to perform Uv‐Vis and emission spectra (10^−4^–10^−5^ M), and therefore we discarded these spectroscopic techniques for our studies. On the other hand, the working concentration conditions of cyclic voltammetry (1 mM), allow significant formation of the host‐guest complexes for the cases of **4**@**3** and **5**@**3**, as can be confirmed from the speciation profiles resulting from the NMR titrations (see supplementary information file). Electrochemistry studies constitute very efficient tool for providing information about the changes in the magnitudes of the intermolecular forces between guest and host upon gain or loss of an electron by either of the components of the host‐guest complex.^[17]^ In fact, redox processes can be used to control de binding strength between two interacting processes, and the term ‘redox‐switch binding’ is used in reference to the change of affinity between a substrate and a host receptor upon its oxidation or reduction.^[17d]^ To our knowledge, the only example of an effective redox‐switchable metallo‐tweezer was reported by Sallé, Goeb and co‐workers recently.^[18]^

**Figure 3:**
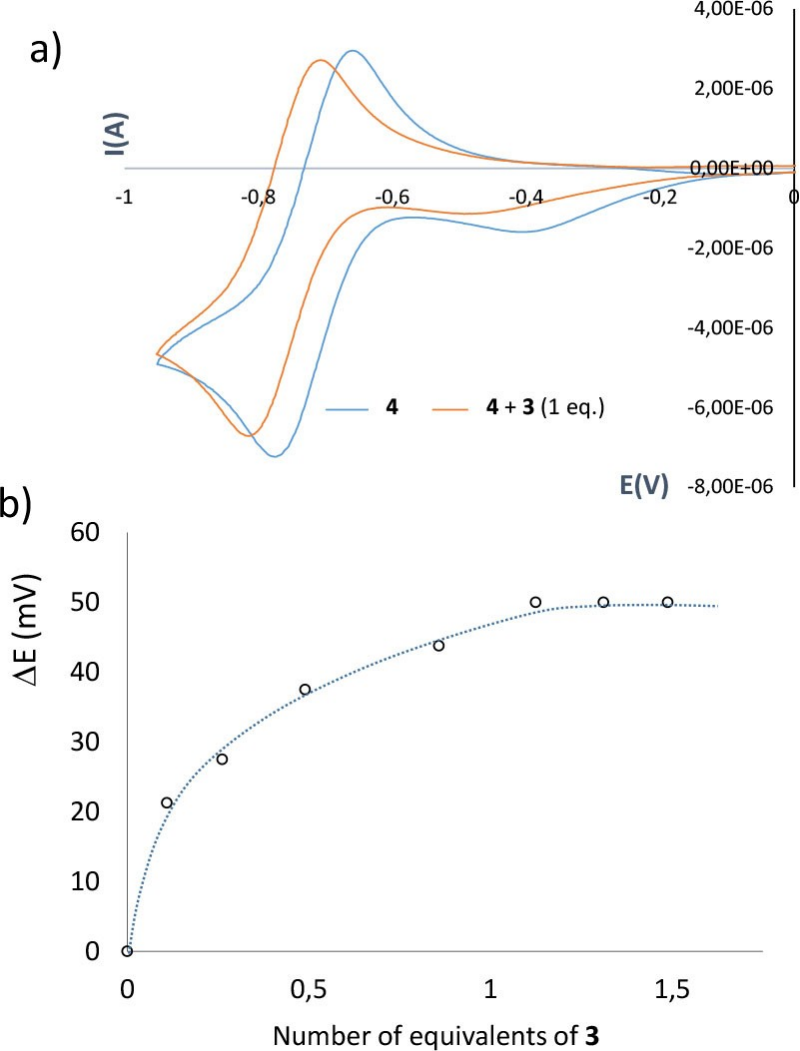
a) Cyclic voltammograms of **4** (1 mM) in the absence and in the presence of **3** (1 mM) in CH_2_Cl_2_. Measurements performed at 100 mV s^−1^ and referenced to ferrocenium/ferrocene (0.46 V). b) Plot of ΔE values of **4** upon incremental amounts of **3**.

NTCDI (**4**) shows one quasi‐reversible reduction wave between 0‐(−1) V, prior to the irreversible reduction of **3**, which occurs at −1.42 V. Upon addition of the metallotweezer **3** to a CH_2_Cl_2_ solution of NTCDI the reduction wave retains its reversible shape, but is shifted gradually to more negative values (see Figure 3). This shift reaches a maximum of −50 mV upon addition of one equivalent of **3**. This cathodic shift is in agreement with the lower tendency of the electrondeficient guest to be reduced in an electrondonating environment, and also reveals that the host has a significant thermodynamic preference for the neutral guest over the electrochemically generated negatively charged NTCDI^−^. In addition, a small decrease of the peak current is also observed, as a consequence of the formation of NTCDI@**3** (**4**@**3**), which diffuses more slowly than free NTCDI. The maximum potential shift can be related to the ratio of the binding constants of the oxidized (*K*_ox_) and reduced (*K*_red_) states as given in Eq. 1,^[19]^ which in our case indicates that the K_red_/K_ox_ ratio is 0.011 (K_red_=23 M^−1^) strongly suggesting that the guest is fully released upon one electron reduction.

(1) $$K{}_{\mathrm{red}}/K{}_{\mathrm{ox}}=\exp\left(\mathrm{nF}\Delta E{}_{1/2}/\mathrm{RT}\right)$$

## Conclusions

In summary, we have shown that the di‐gold metallotweezer **3**, serves as a selective receptor for electron‐deficient aromatic substrates. The ability of the tweezer for trapping a planar pincer‐Au(III) complex (**8**) has also been demonstrated. The encapsulating properties of the receptor are justified by π‐π attractive interactions, although we also showed that hydrogen bonding plays an important role in the case of the encapsulation of the nitro‐containing molecule TNFLU. A remarkable feature of this molecular tweezer is that it can adapt its shape to maximize the face‐to‐face overlap with the surface of the guest, as shown by the approach of the two pyrene moieties that constitute the tweezer arms from the 10 Å of the empty tweezer, to 7 Å in the case of the tweezer with an encapsulated planar guest. This guest‐induced fit distortion allows that the pyrene moieties of the receptor are disposed at a quasi‐parallel orientation and establish the optimum distance for an effective π‐π‐stacking interaction with the guest molecule.

Finally, the encapsulation of the redox‐active species NTCDI can be controlled electrochemically, as it displays a large binding affinity with the molecular tweezer **3** in its neutral state, but it is released upon one electron reduction, thus constituting an interesting example of redox‐switchable binding.

## Conflict of interest

The authors declare no conflict of interest.

## Supporting information

As a service to our authors and readers, this journal provides supporting information supplied by the authors. Such materials are peer reviewed and may be re‐organized for online delivery, but are not copy‐edited or typeset. Technical support issues arising from supporting information (other than missing files) should be addressed to the authors.

Supplementary
