# Supplementary material for: Shape‐Adaptability and Redox‐Switching Properties of a Di‐Gold Metallotweezer
Source: Chemistry. 2021 May 21;27(37):9661–5. doi: 10.1002/chem.202100794 (PMC8362111; doi:10.1002/chem.202100794)
Supplement: Supplementary file 1 — Supplementary [file CHEM-27-9661-s001.pdf]

Chemistry–A European Journal

Supporting Information

Shape-Adaptability and Redox-Switching Properties of a Di-Gold Metallotweezer

Susana Ibáñez* and Eduardo Peris*

Supporting Information

**General considerations**	**S1**
**1. Synthesis of 6**	**S1**
**2. Spectroscopic data**	**S3**
2.1. ^{1}H and ^{13}C NMR spectra of **6** in $CDCl_3$	S3
**3. X-Ray crystallography**	**S4-S5**
**4. Titration experiments**	**S6-S14**
4.1. ^{1}H NMR titration experiments	S6-S14
**5. DOSY experiment**	**S15-S16**
**6. High resolution mass spectra**	**S17-S18**
**7. Cyclic voltammetry studies**	**S18**
**8. References**	**S19**

General considerations. The carbazolyl-linked complex **3**,[1] N,N'-dimethyl-naphthalenetetracarboxy diimide(**4**),[2] and Au(C^N^C)(C≡CC_6H_4-OCH_3-*p*)(**8**)[3] were prepared according to literature methods. All other reagents were used as received from commercial suppliers. NMR spectra were recorded on a Bruker 400 MHz using $CDCl_3$ as solvents. High Resolution Mass Spectra (HRMS) were recorded on a Q-TOF Premier mass spectrometer (Waters) with an electrospray source operating in the V-mode. Nitrogen was used as the drying and cone gas at flow rates of 300 and 30 Lh^{-1}, respectively. The temperature of the source block was set to 120ºC, and the desolvation temperature was set to 150ºC. Capillary voltage of 3.5 kV was used in the positive scan modes and the cone voltage was adjusted typically to 20 V. Mass calibration was performed by using solutions of NaI in isopropanol/water (1:1) from *m/z* 50 to 3000. Elemental analyses were carried out on a TruSpec Micro Series. Infrared spectra (FTIR) were performed on a FT/IR-6200 (Jasco) spectrometer with a spectral window of 4000-600 cm^{-1}. The BindFitv0.5 program was employed for the calculation of the association constants. UV/Visible absorption spectra were recorded on a Varian Cary 300 BIO spectrophotometer using toluene under ambient conditions. Emission spectra were recorded on a modular Horiba FluoroLog-3 spectrofluorometer employing degassed toluene. The electrochemical studies were carried out by using an Autolab Potentiostat (Model PGSTAT101) using a three-electrode cell. The cell was equipped with platinum working and counter electrodes, as well as a silver wire reference electrode. In all experiments, a 0.1 M solution of $[NBu_4][PF_6]$ in CH_2Cl_2 was used as the supporting electrolyte with analyte concentration of 1 mM. Measurements were performed at 100 mVs^{-1} scan rates. All redox potentials were referenced to ferrocenium/ferrocene (Fc/Fc^+; $E_{1/2}$ = 0.46 V (CH_2Cl_2) *vs*. SCE)[4] as internal standard.

Synthesis of 6. 2,4,7-trinitro-9-fluorenone (TNFLU, **5**) (105.9 mg, 0.336 mmol) and NaOH (33.50 mg, 0.840 mmol) were dissolved in deoxygenated methanol (40 mL). The solution was heated at reflux for 3 hours. The mixture was evaporated to dryness and the solid residue was extracted with dichloromethane, and the solution was filtered through a short pad of Celite. **6** was isolated as orange solid. Yield: 82.90 mg, 82%. Anal. Calcd. for $C_{14}H_8N_2O_6$ (300.224): C, 56.00; H, 2.69; N, 9.33. Found: C, 56.09; H, 2.77; N, 9.03. ^{1}H NMR (300 MHz, $CDCl_3$): δ 8.53 (d, $^3J_{H-H}$ = 3.0 Hz, 1H, C*H*), 8.46 (dd, $^3J_{H-H}$ = 9.0 Hz, $^3J_{H-H}$ = 3.0 Hz, 1H, C*H*), 8.22 (d, $^3J_{H-H}$ = 3.0 Hz, 1H, C*H*), 8.15 (d, $^3J_{H-H}$ = 9.0 Hz, 1H,

CH), 8.05 (d, $^{3}J_{H-H}$ = 3.0 Hz, 1H, CH), 4.18 (s, 3H, OCH_3). ^{13}C {^{1}H} NMR (75 MHz, $CDCl_3$): δ 188.83 ($C_{q\ C≡O}$), 156.40 (C_q), 150.95 (C_q), 149.05 (C_q), 146.97 (C_q), 136.96 (C_q), 135.24 (C_q), 134.83 (C_q), 130.50 (CH), 125.94 (CH), 119.77 (CH), 113.28 (CH), 112.35 (CH), 56.96 (O$C$$H_3$).

2. Spectroscopic data

2.1. ^{1}H and ^{13}C NMR spectra of 6 in $CDCl_3$

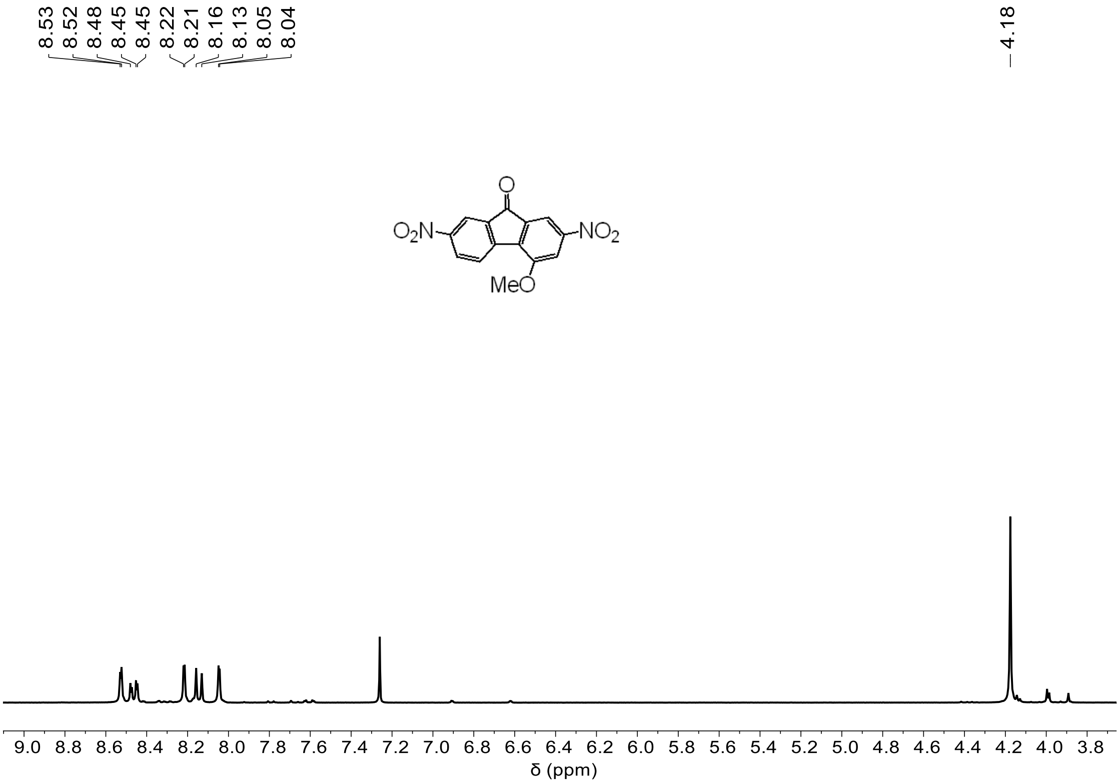

Figure S1. ^{1}H NMR spectrum of **3** in $CDCl_3$

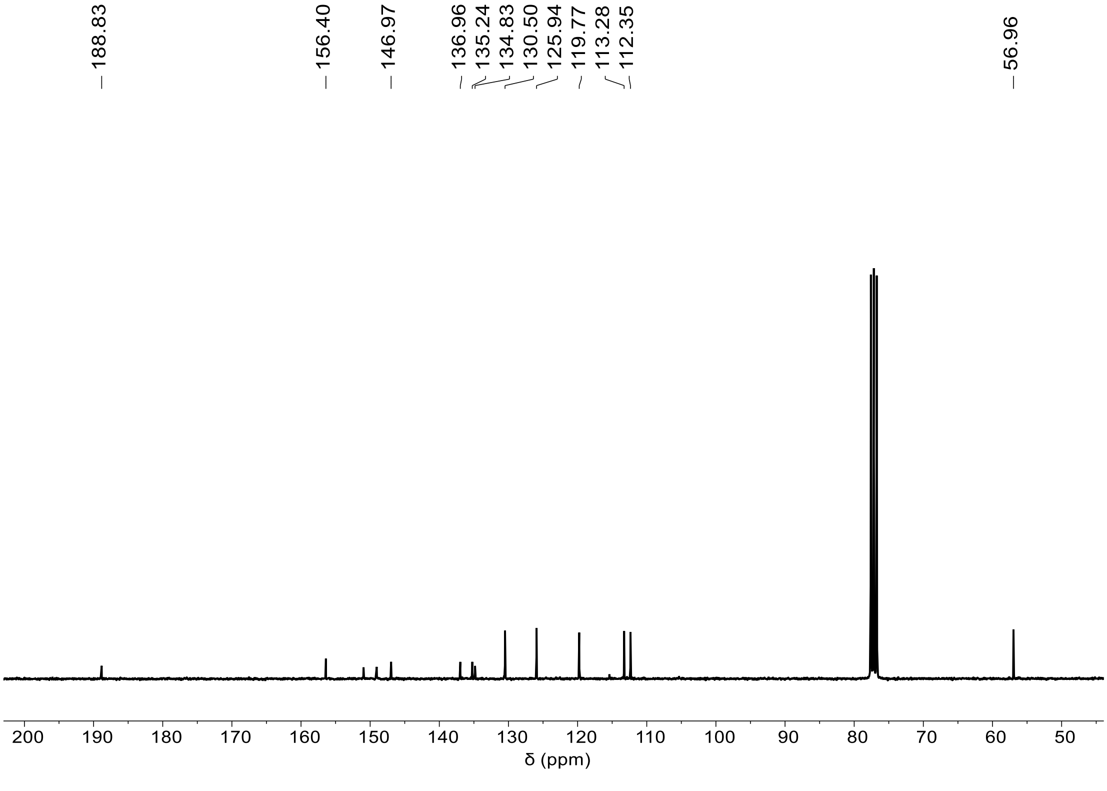

Figure S2. ^{13}C NMR spectrum of **3** in $CDCl_3$

3. X-Ray Crystallography

X-Ray Diffraction studies for complexes 4@3 and 6@3.

Crystals suitable for X-Ray studies of complexes **4@3** and **6@3** were obtained by slow diffusion of hexane into a concentrated solution of the complex in chloroform. Diffraction data of complexes were collected on an Agilent SuperNova diffractometer equipped with an Atlas CCD detector using Cu-Kα radiation (λ = 1.54184 Å). Single crystals were mounted on a MicroMount® polymer tip (MiteGen) in a random orientation. Absorption corrections based on the multi-scan method were applied. Using Olex2,[5] the structure of the two complexes was solved using ShelXS[6] and refined with ShelXL[7] refinement package using Least Squares minimisation. Key details of the crystals and structure refinement data are summarized in Supplementary Table S1. Further crystallographic details can be found in the CIF files, which were deposited at the Cambridge Crystallographic Data Centre, Cambridge, UK. The reference number for complex **4@3** and **6@3** were assigned as 2065669 and 2065771.

Table S1. Summary of crystal data, data collection, and structure refinement details

	**complex 4@3**	**complex 6@3**
Empirical formula	$C_{107}H_{117}Au_2Cl_3N_7O_4$	$C_{121}H_{126}Au_2Cl_9N_9O_{12}$
Formula weight	2065.35	2611.28
Temperature/K	180(40)	200.00(14)
Crystal system	monoclinic	triclinic
Space group	C2/c	P-1
a/Å	18.7085(2)	16.34826(15)
b/Å	21.9168(2)	20.75317(19)
c/Å	25.2197(3)	20.8290(2)
α/°	90	81.7912(8)
β/°	107.2730(10)	67.8226(9)
γ/°	90	72.4604(8)
Volume/Å^3	9874.48(19)	6236.42(11)
Z	4	2
$\rho_{calc}g/cm^3$	1.389	1.391
μ/mm^{-1}	6.669	6.610
F(000)	4196.0	2644.0
Crystal size/mm^3	0.312× 0.206 × 0.185	0.293 × 0.191 × 0.133
2Θ range for data collection	7.342 to 143.402	8.452 to 133.198

Index ranges	$-19 \leq h \leq 23$, $-26 \leq k \leq 26$, $-29 \leq l \leq 31$	$-19 \leq h \leq 19$, $-24 \leq k \leq 24$, $-24 \leq l \leq 24$
Reflections collected	53215	127795
Independent reflections	9600 [R_{int} = 0.0280, R_{sigma} = 0.0145]	22007 [R_{int} = 0.0552, R_{sigma} = 0.0262]
Data/restraints/ parameters	9600/598/615	220107/1338/1505
Goodness-of-fit on F^2	1.079	1.035
Final R indexes [I>=2σ (I)]	R_1 = 0.0484, wR_2 = 0.1488	R_1 = 0.0619, wR_2 = 0.1835
Final R indexes [all data]	R_1 = 0.0490, wR_2 = 0.1495	R_1 = 0.0716, wR_2 = 0.1981
Largest diff. peak/hole / e $Å^{-3}$	1.45/-1.10	2.82/-1.40

4. Titration experiments

4.1. ^{1}H NMR titration experiments

The recognition capability of complex **3** (host) was studied by ^{1}H NMR titration experiments, by adding increasing amounts of NTCDI (**4**), or TNFLU (**5**), or 2,7-dinitro-4-methoxy-fluorenone (**6**), or (Au(C^N^C)(C≡CC$_6$H$_4$-OCH$_3$-*p*) (**8**) (guest) to a solution of complex **2** (host). The experiments were carried out in CDCl$_3$ or toluene-d$_8$, at constant concentrations of the host (0.5 mM). Two solutions were prepared: solution A (containing only host at 0.5 mM) and solution B (containing host at 0.5 mM and guest at different concentration). The addition of increasing amounts of solution B to solution A produced a perturbation of some of the proton resonances of the host. The association constant was calculated by least-square analysis, by using the BindFitv0.5 program.

Titration of 3 with NTCDI in CDCl$_3$.

Table S2. Data values from the titration study of complex **3** with **NTCDI**.

**[3]** M	[**4**] M	δ_{NH}	δ_{CH}	δ_{CH}	δ_{CH}	δ_{NCH2}	equiv. **4**
0,00054453	0	9,08	8,59	8,19	8,04	5,27	0
0,00054453	6,4166E-05	9,09	8,57	8,17	8,01	5,26	0,1
0,00054453	0,00012587	9,09	8,56	8,16	7,99	5,25	0,2
0,00054453	0,00018524	9,09	8,54	8,14	7,98	5,24	0,3
0,00054453	0,00024241	9,1	8,53	8,13	7,96	5,23	0,4
0,00054453	0,00035062	9,1	8,51	8,12	7,94	5,22	0,6
0,00054453	0,00045138	9,11	8,49	8,1	7,92	5,21	0,8
0,00054453	0,00063339	9,11	8,46	8,07	7,89	5,2	1,2
0,00054453	0,000935	9,12	8,41	8,03	7,83	5,17	1,7
0,00054453	0,00117474	9,12	8,37	8	7,79	5,14	2,2
0,00054453	0,00141312	9,12	8,35	7,98	7,76	5,13	2,6
0,00054453	0,00163625	9,12	8,35	7,98	7,76	5,13	3,0

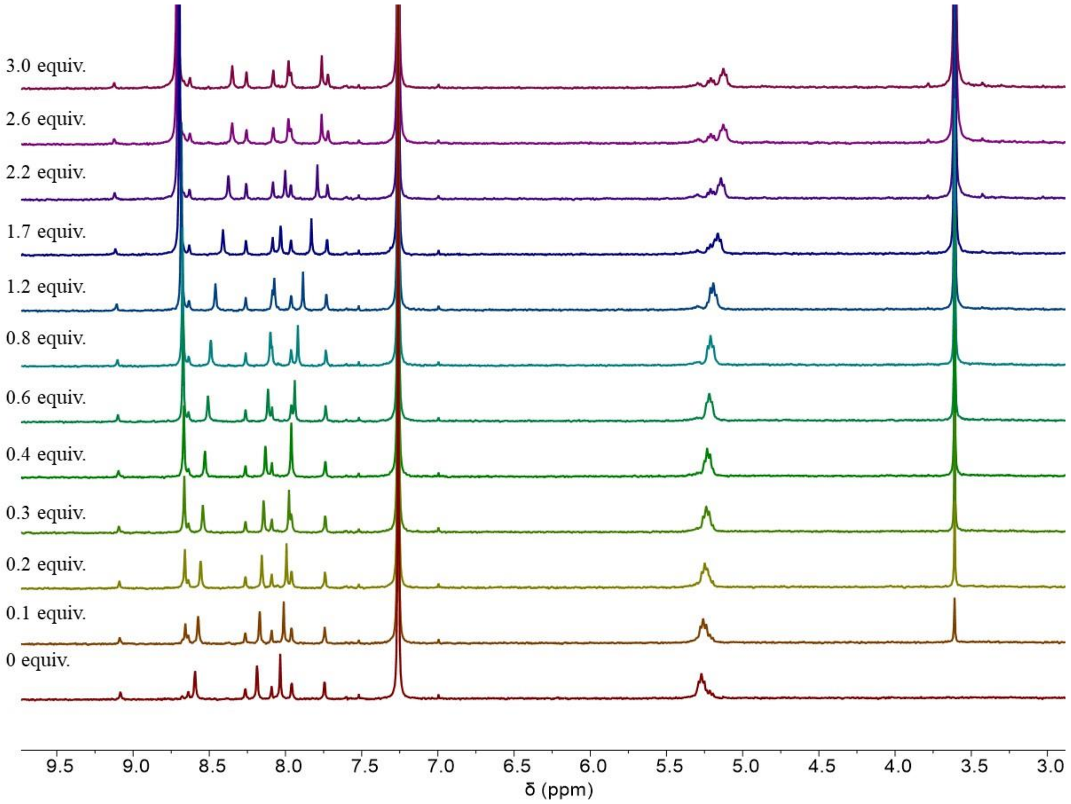

Figure S3. Selected region and spectra of the titration of complex **3** with NTCDI.

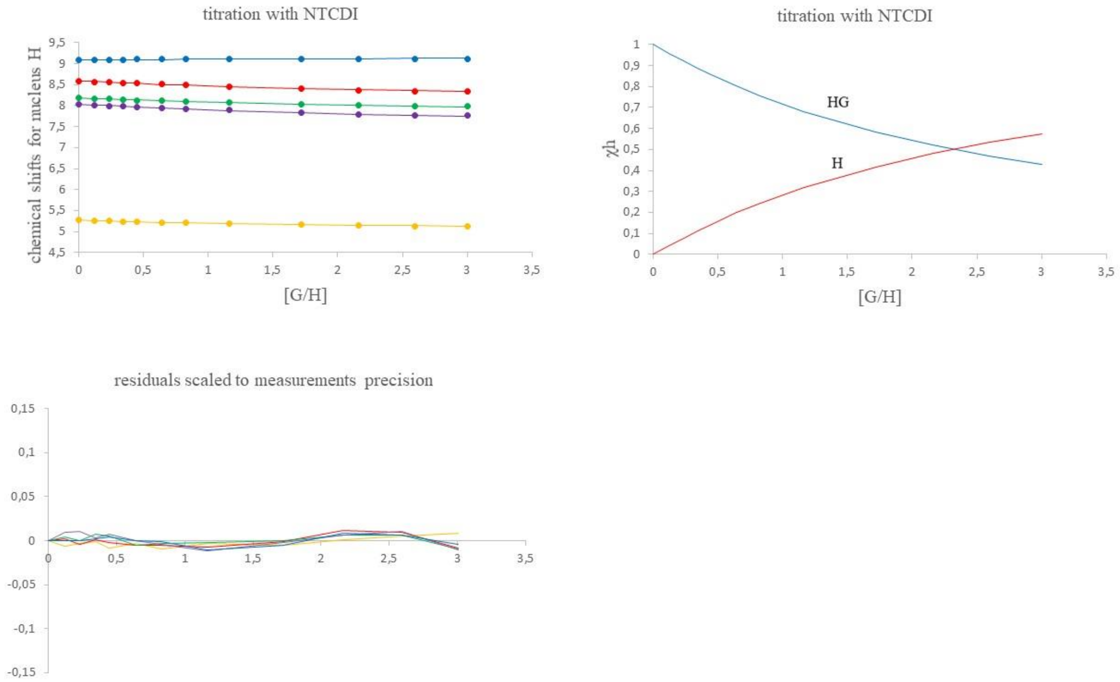

Figure S4. Non-linear least-squares fitting of the chemical shift changes of H during titration experiments of **3** with NTCDI. The Figure on the left represents the speciation profiles.

Titration of 3 with TNFLU in $CDCl_3$.

Table S3. Data values from the titration study of complex **3** with TNFLU.

[**3**] M	[**5**] M	δ_{NH}	δ_{CH}	δ_{CH}	δ_{CH}	δ_{NCH2}	equiv. **5**
0,00052437	0	9,08	8,6	8,19	8,04	5,27	0
0,00052437	0,00012565	9,21	8,54	8,16	7,99	5,22	0,2
0,00052437	0,00024884	9,32	8,5	8,13	7,95	5,17	0,5
0,00052437	0,00036963	9,42	8,46	8,1	7,92	5,13	0,7
0,00052437	0,00048811	9,5	8,43	8,09	7,89	5,09	0,9
0,00052437	0,00071834	9,59	8,39	8,06	7,85	5,05	1,4
0,00052437	0,00094006	9,66	8,36	8,04	7,82	5,02	1,8
0,00052437	0,00135972	9,75	8,31	8,01	7,78	4,97	2,6
0,00052437	0,00175045	9,82	8,28	7,99	7,75	4,94	3,3
0,00052437	0,00245628	9,89	8,22	7,96	7,71	4,9	4,7
0,00052437	0,00362593	9,95	8,19	7,93	7,66	4,85	6,9
0,00052437	0,00455566	9,96	8,17	7,92	7,64	4,83	8,7
0,00052437	0,0054801	9,96	8,15	7,91	7,63	4,82	10,4
0,00052437	0,00634538	9,96	8,15	7,91	7,63	4,82	12,1

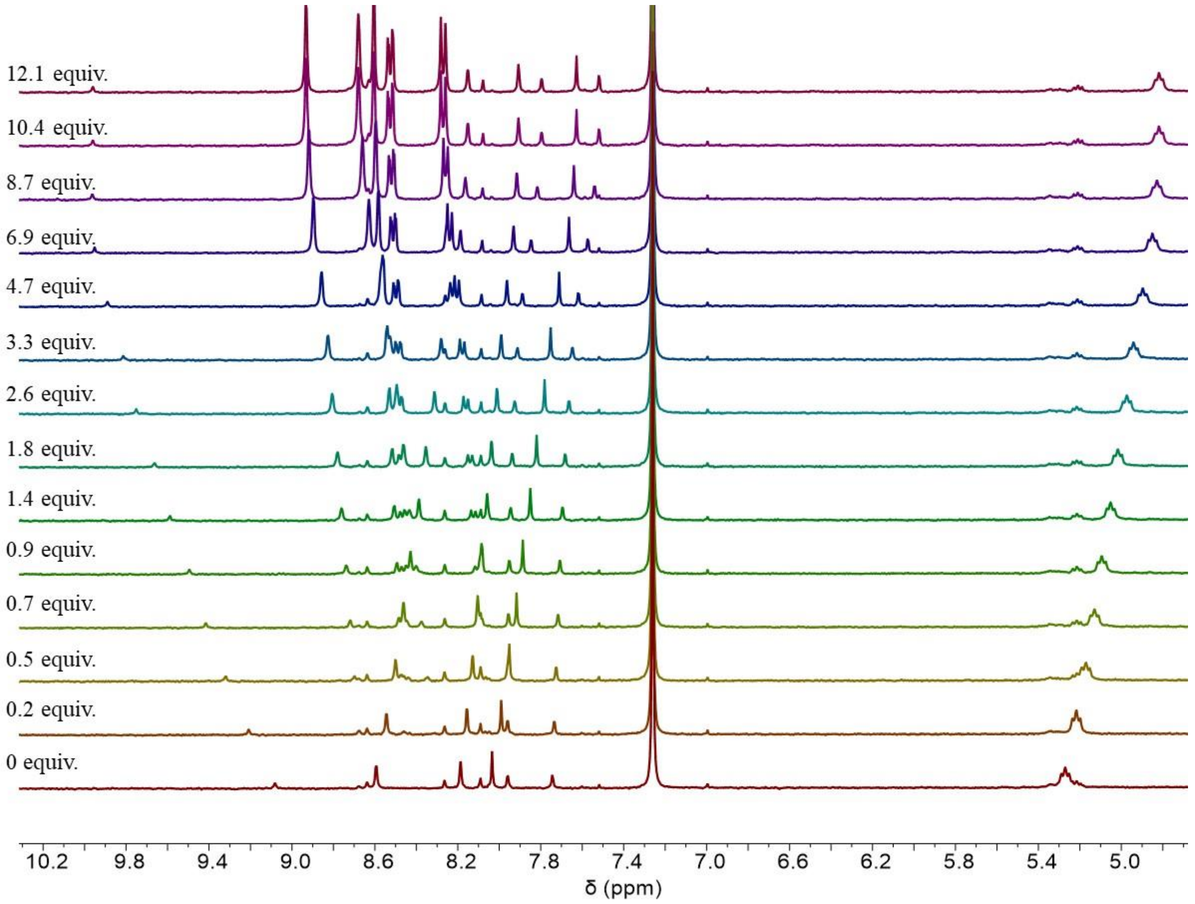

Figure S5. Selected region and spectra of the titration of complex **3** with TNFLU.

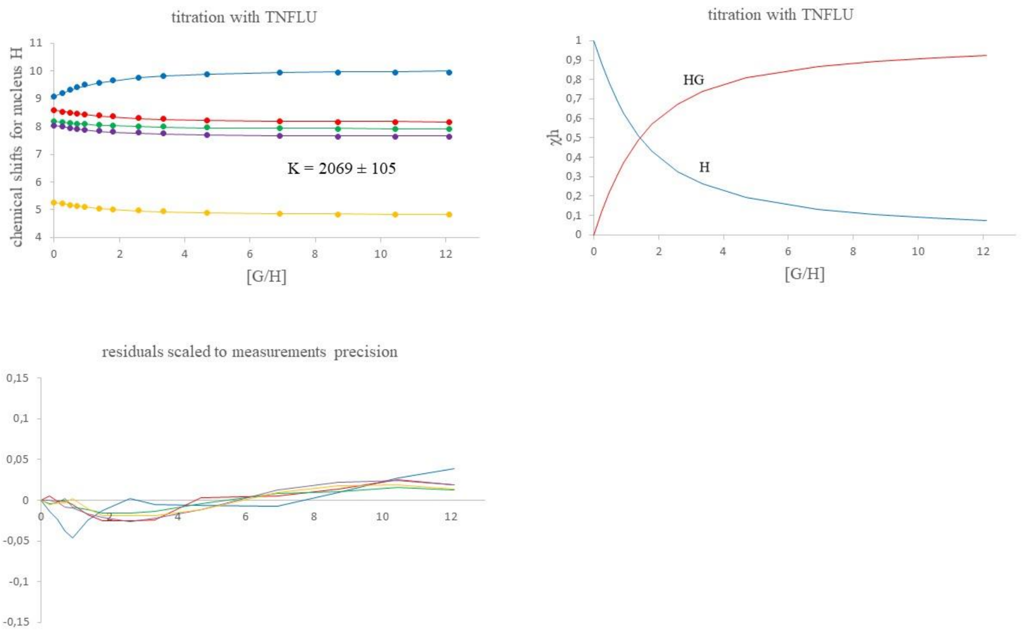

Figure S6. Non-linear least-squares fitting of the chemical shift changes of H during titration experiments of **3** with TNFLU. The Figure on the left represents the speciation profiles.

Titration of 3 with 2,7-dinitro-4-methoxy-fluorenone in $CDCl_3$.

Table S4. Data values from the titration study of complex **3** with 2,7-dinitro-4-methoxy-fluorenone.

**[3]** M	[**6**] M	δ_{NH}	δ_{CH}	δ_{CH}	δ_{CH}	δ_{NCH2}	equiv. **6**
0,00054453	0	9,08	8,6	8,19	8,04	5,27	0
0,00054453	0,00019351	9,1	8,58	8,18	8,03	5,26	0,4
0,00054453	0,00037958	9,11	8,57	8,17	8,01	5,24	0,7
0,00054453	0,00073105	9,13	8,55	8,16	8	5,23	1,3
0,00054453	0,00105741	9,14	8,53	8,15	7,99	5,21	1,9
0,00054453	0,00164486	9,17	8,5	8,13	7,96	5,18	3,0
0,00054453	0,00261243	9,21	8,47	8,1	7,93	5,13	4,8
0,00054453	0,0033763	9,24	8,43	8,09	7,91	5,1	6,2
0,00054453	0,00426169	9,26	8,41	8,07	7,88	5,07	7,8
0,00054453	0,00493459	9,27	8,4	8,06	7,87	5,06	9,1

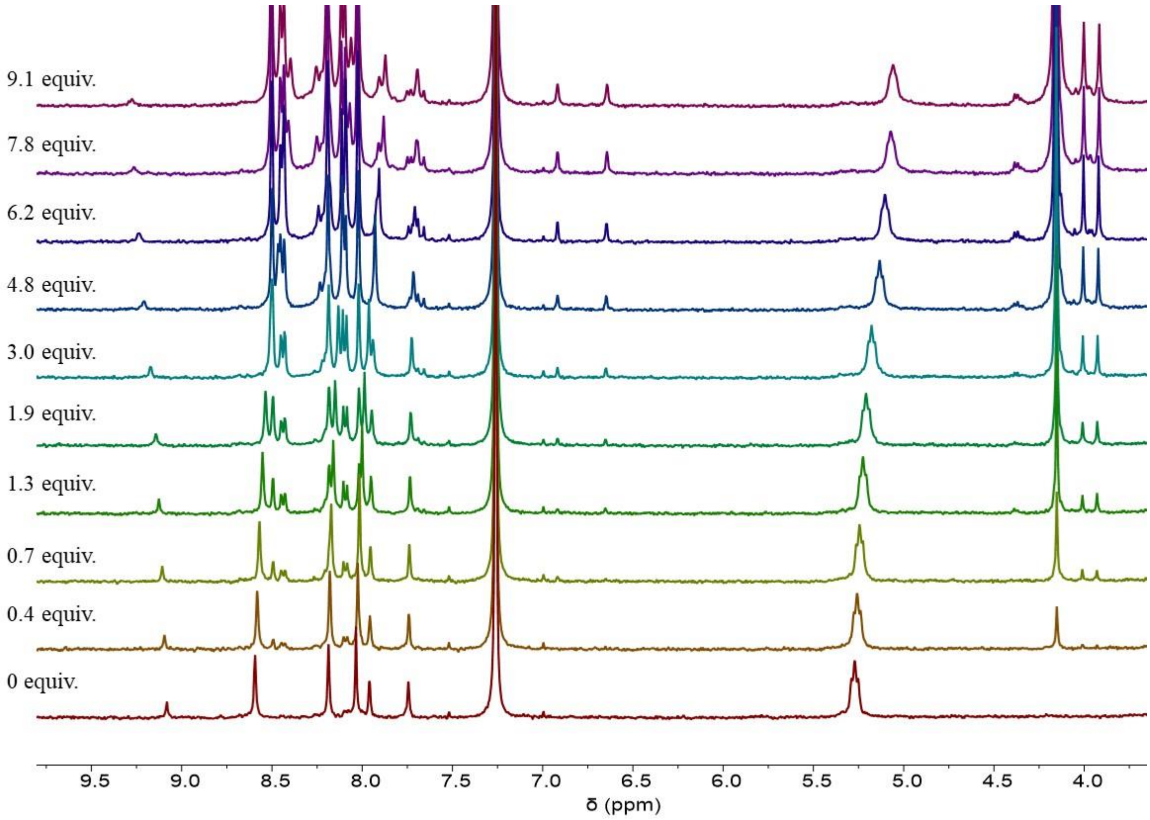

Figure S7. Selected region and spectra of the titration of complex **3** with 2,7-dinitro-4-methoxy-fluorenone.

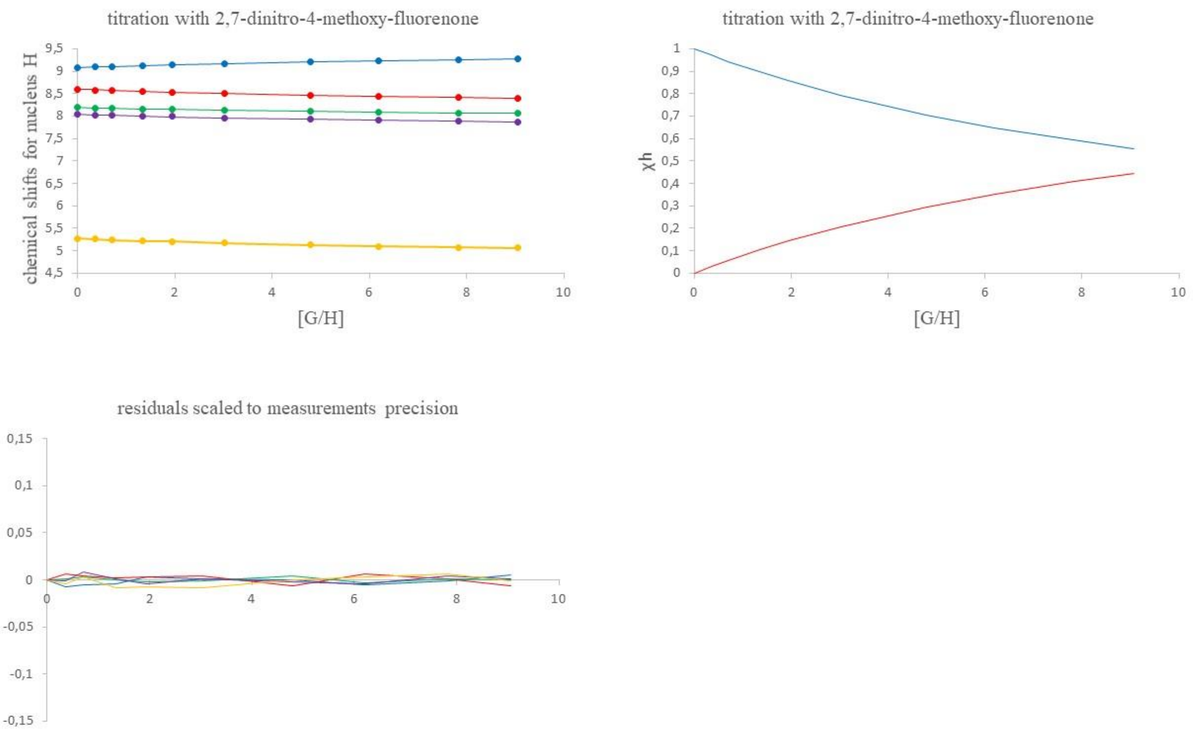

Figure S8. Non-linear least-squares fitting of the chemical shift changes of H during titration experiments of **3** with 2,7-dinitro-4-methoxy-fluorenone. The Figure on the left represents the speciation profiles.

Titration of 3 with Au(C^N^C)(C≡CC$_6$H$_4$-OCH$_3$-p) in CDCl$_3$.

Table S5. Data values from the titration study of complex **3** with Au(C^N^C)(C≡CC$_6$H$_4$-OCH$_3$-*p*).

[**3**] M	[**8**] M	δ_{CH}	δ_{CH}	δ_{CH}	δ_{CH}	δ_{NCH2}	equiv. **8**
0,00052437	0	9,09	8,59	8,19	8,04	5,27	0
0,00052437	0,00032364	9,09	8,58	8,17	8,02	5,26	0,6
0,00052437	0,00063483	9,1	8,57	8,16	8,01	5,25	1,2
0,00052437	0,00093428	9,1	8,56	8,14	7,99	5,24	1,8
0,00052437	0,00122264	9,1	8,55	8,13	7,98	5,23	2,3
0,00052437	0,00150052	9,11	8,54	8,12	7,97	5,22	2,9
0,00052437	0,00176847	9,11	8,53	8,11	7,96	5,21	3,4
0,00052437	0,00227665	9,11	8,52	8,09	7,94	5,2	4,3
0,00052437	0,00275095	9,12	8,51	8,07	7,92	5,18	5,2
0,00052437	0,00361062	9,12	8,49	8,05	7,9	5,16	6,9

0,00052437	0,00436915	9,13	8,47	8,03	7,88	5,15	8,3
0,00052437	0,00564668	9,14	8,44	7,99	7,84	5,12	10,8
0,00052437	0,00668087	9,15	8,42	7,97	7,81	5,1	12,7
0,00052437	0,00825284	9,15	8,42	7,96	7,81	5,1	15,7

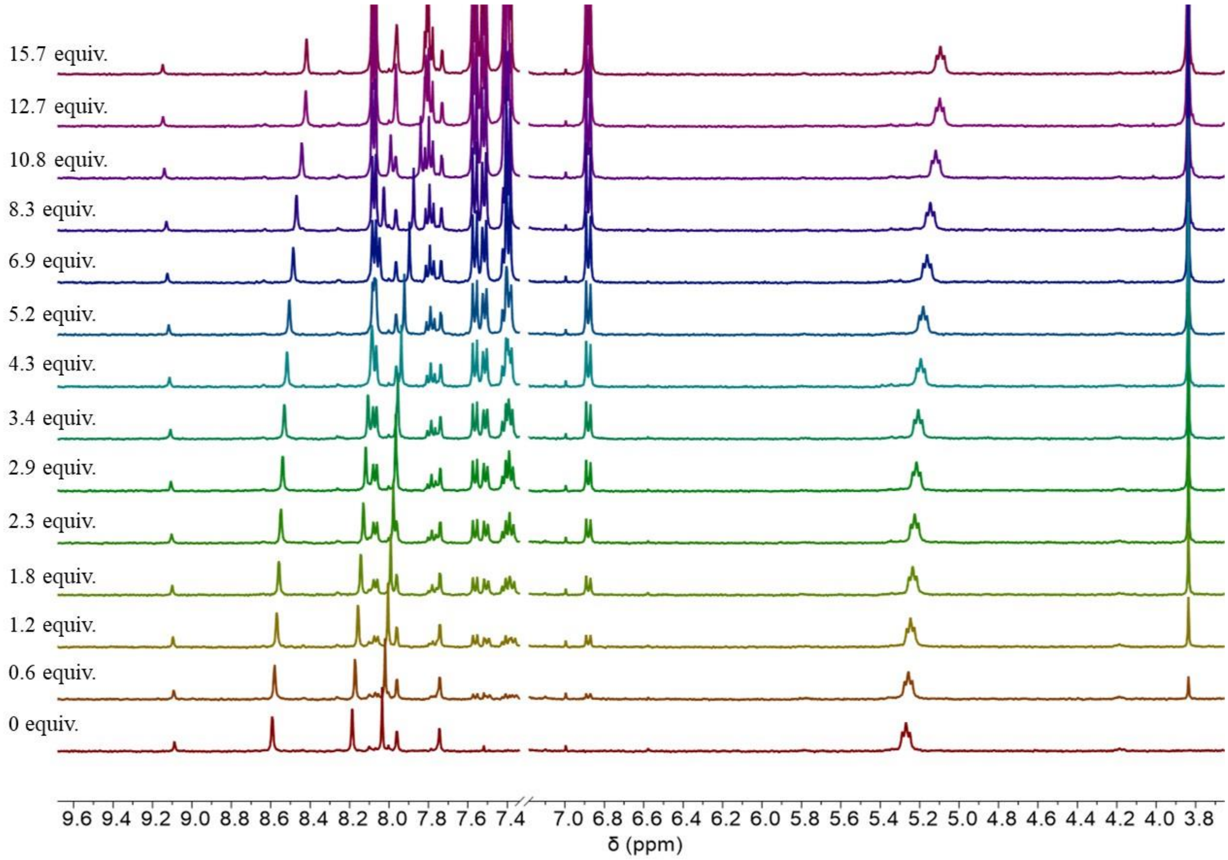

Figure S9. Selected region and spectra of the titration of complex **3** with Au(C^N^C)(C≡CC_6H_4-OCH_3-*p*).

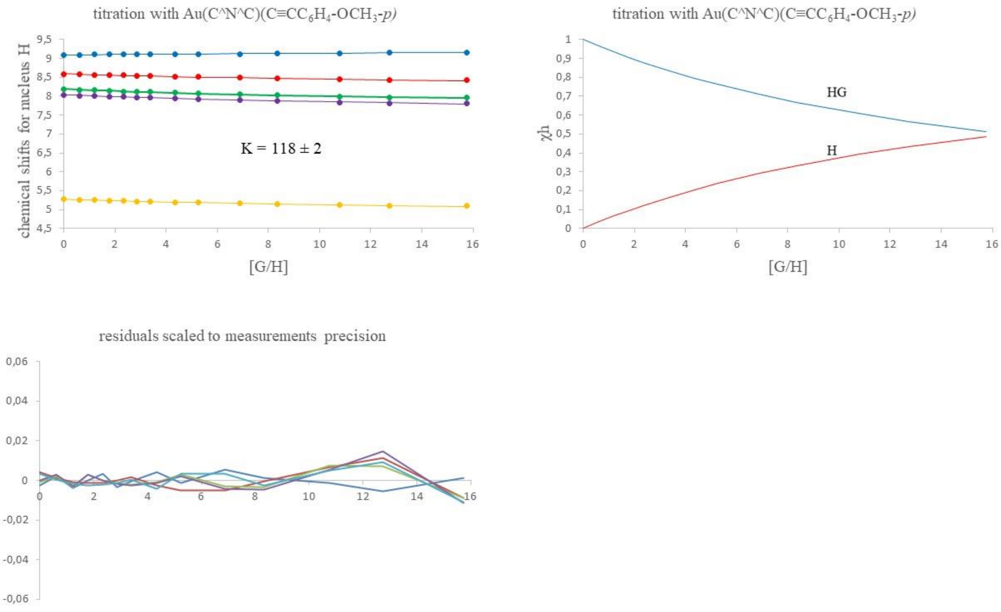

Figure S10. Non-linear least-squares fitting of the chemical shift changes of H during titration experiments of **3** with Au(C^N^C)(C≡CC$_6$H$_4$-OCH$_3$-*p*). The Figure on the left represents the speciation profiles.

Titration of 3 with Au(C^N^C)(C≡CC$_6$H$_4$-OCH$_3$-p) in toluene-d$_8$.

Table S6. Data values from the titration study of complex **3** with Au(C^N^C)(C≡CC$_6$H$_4$-OCH$_3$-*p*).

[**3**] M	[**8**] M	δ_{CH}	δ_{CH}	δ_{CH}	equiv. **8**
0,0005042	0	8,36	7,99	7,76	0
0,0005042	0,00028982	8,34	7,97	7,74	0,6
0,0005042	0,00055816	8,33	7,96	7,73	1,1
0,0005042	0,00080734	8,33	7,95	7,72	1,6
0,0005042	0,00103934	8,32	7,94	7,71	2,1
0,0005042	0,00125587	8,32	7,93	7,7	2,5
0,0005042	0,00145843	8,31	7,93	7,7	2,9
0,0005042	0,00182672	8,31	7,92	7,69	3,6
0,0005042	0,00215291	8,31	7,92	7,69	4,3
0,0005042	0,00270494	8,3	7,91	7,68	5,4
0,0005042	0,00315427	8,3	7,91	7,67	6,2
0,0005042	0,00384147	8,29	7,9	7,67	7,6

0,0005042	0,00434232	8,29	7,89	7,66	8,6
0,0005042	0,00502347	8,28	7,89	7,66	10,0

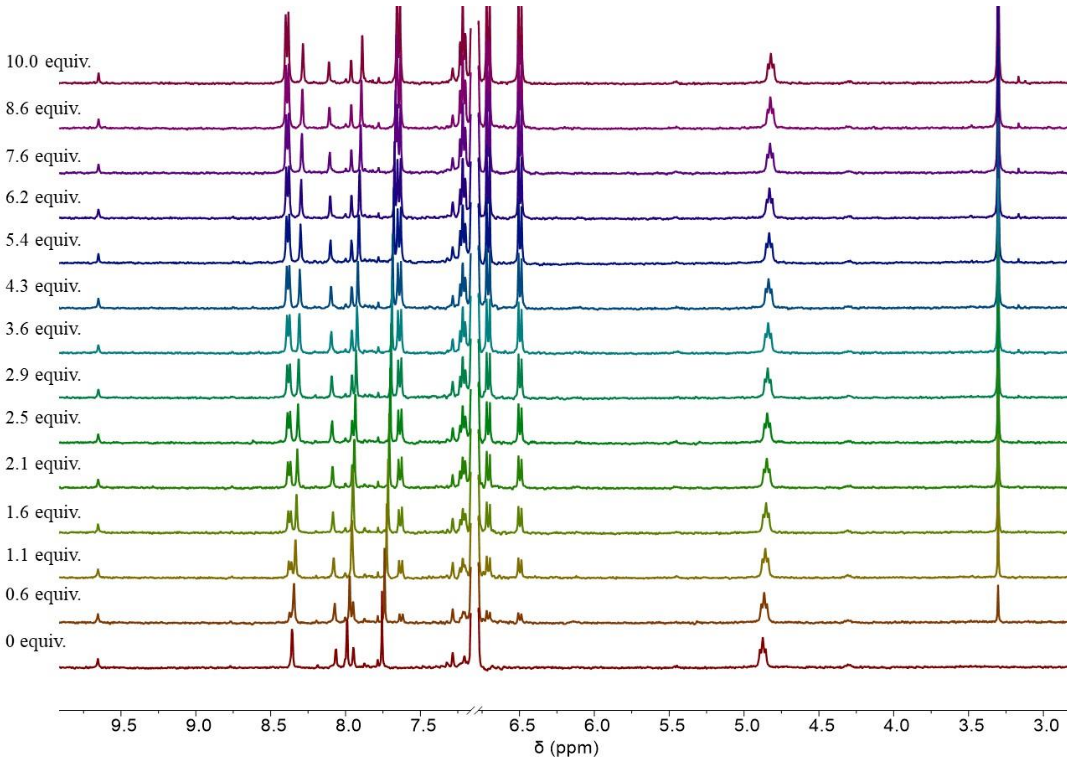

Figure S11. Selected region and spectra of the titration of complex **3** with Au(C^N^C)(C≡CC$_6$H$_4$-OCH$_3$-*p*).

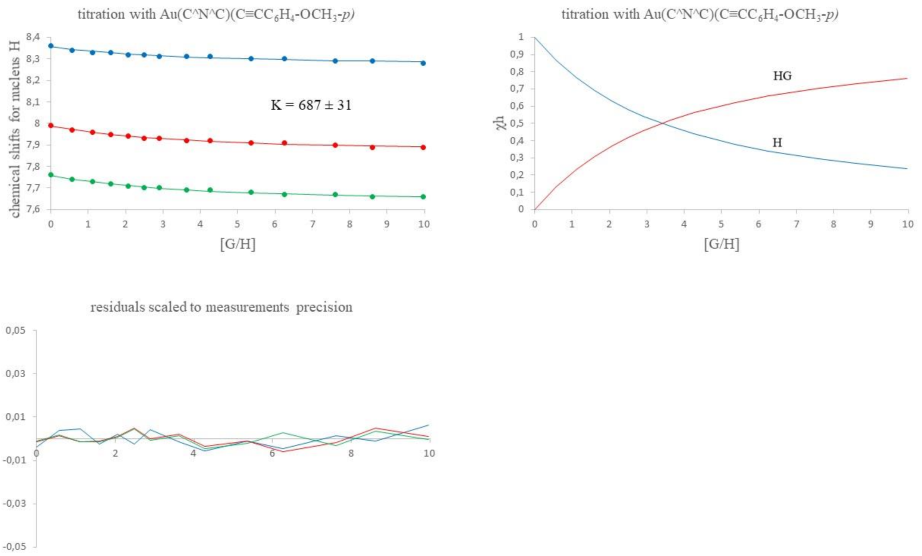

Figure S12. Non-linear least-squares fitting of the chemical shift changes of H during titration experiments of **3** with Au(C^N^C)(C≡CC$_6$H$_4$-OCH$_3$-*p*). The Figure on the left represents the speciation profiles.

5. DOSY experiments

The experiments were carried out in $CDCl_3$, at constant concentrations of 5 mM.

Complexes	G (m^2/s)
**3**	5.59 10^{-10}
**4@3**	5.34 10^{-10}
**5@3**	5.28 10^{-10}

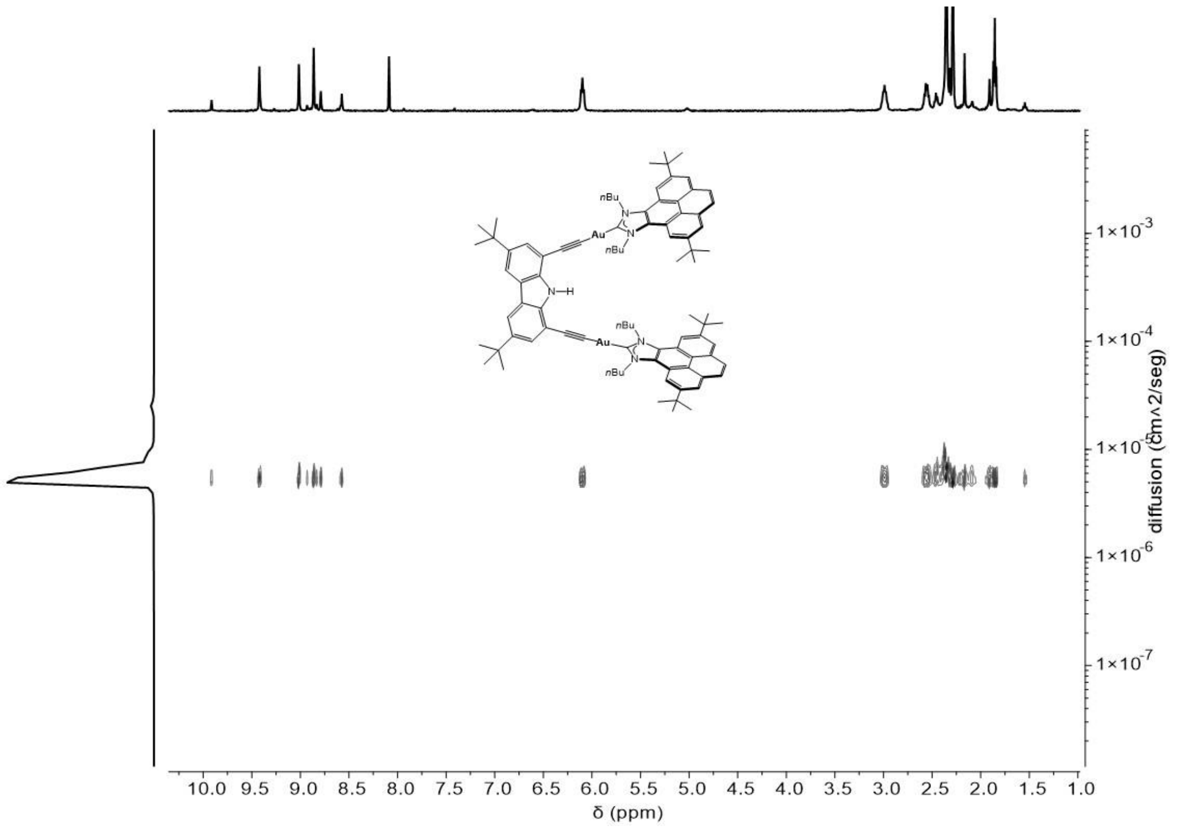

Figure S13. DOSY NMR spectrum of **3**.

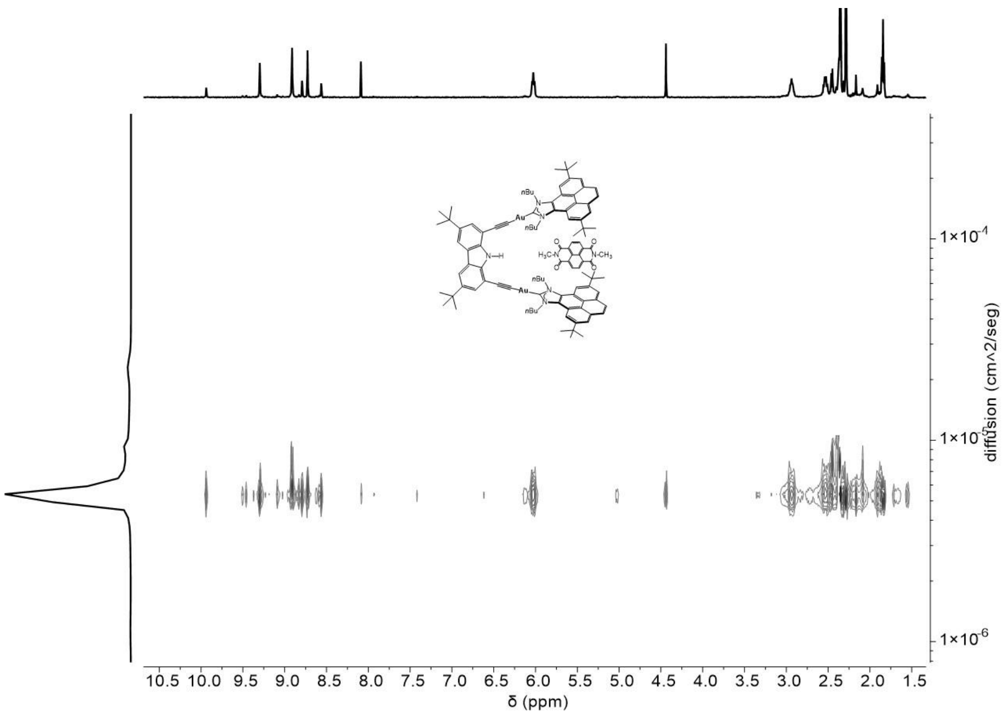

Figure S14. DOSY NMR spectrum of **4@3**.

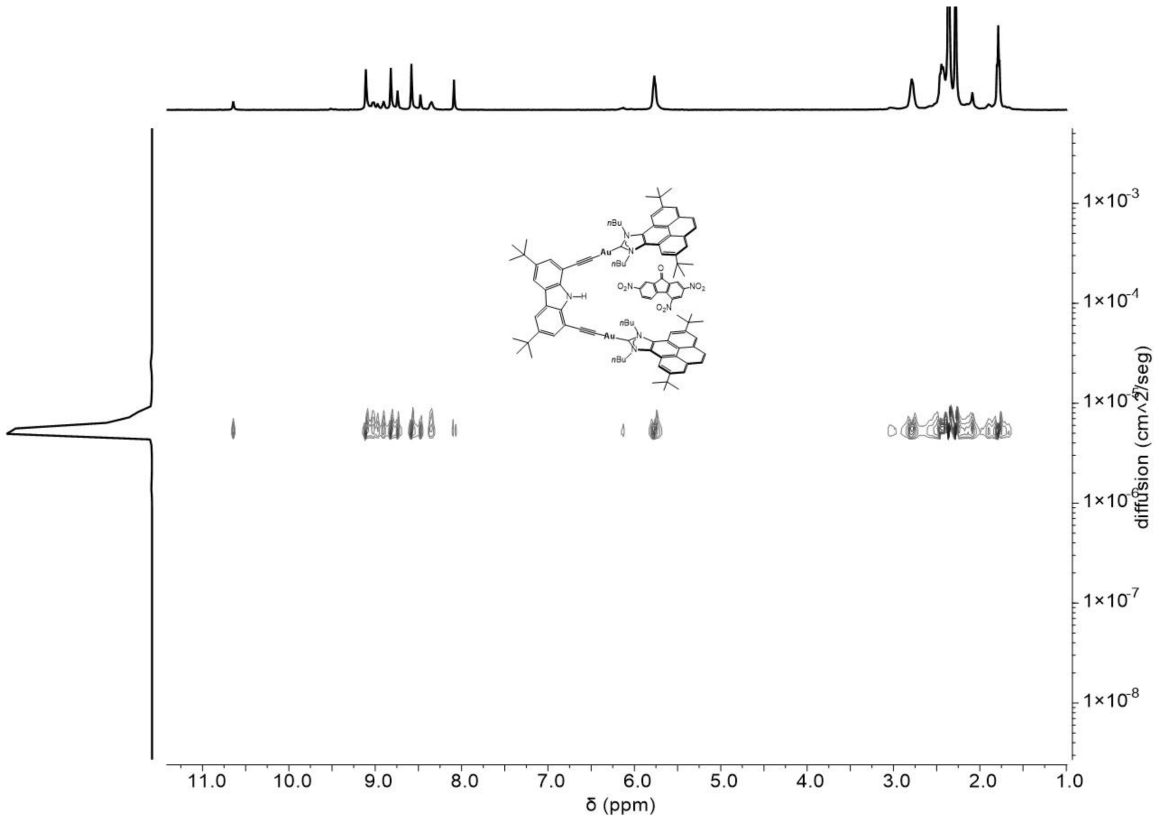

Figure S15. DOSY NMR spectrum of **5@3.**

6.- High resolution mass spectra

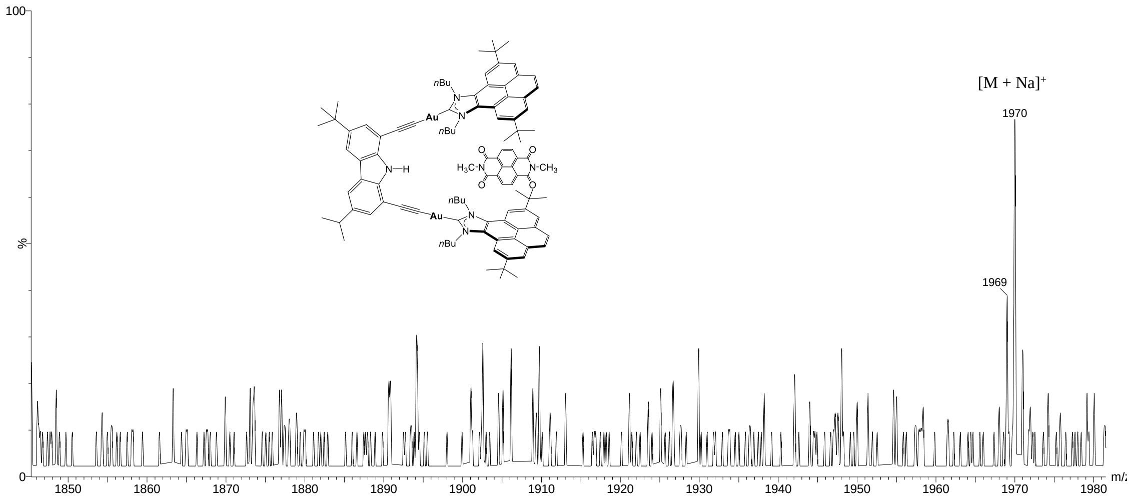

Figure S16. Selected region of the HRMS ESI-TOF-MS (positive mode) spectrum of **4@3**.

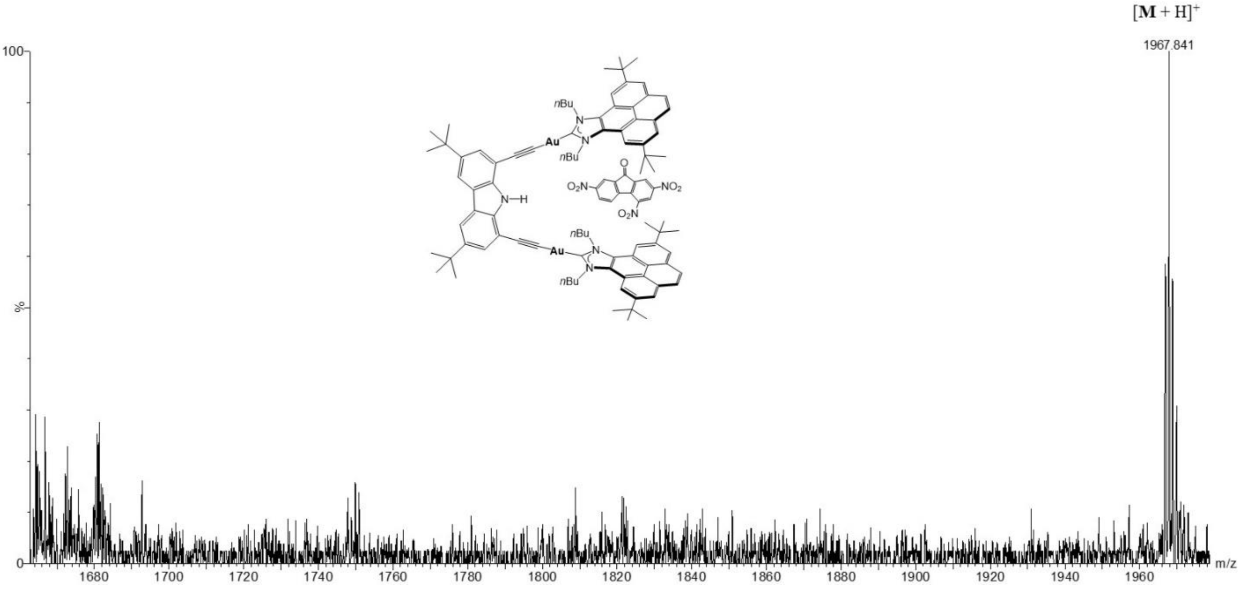

Figure S17. Selected region of the HRMS ESI-TOF-MS (positive mode) spectrum of **5@3**.

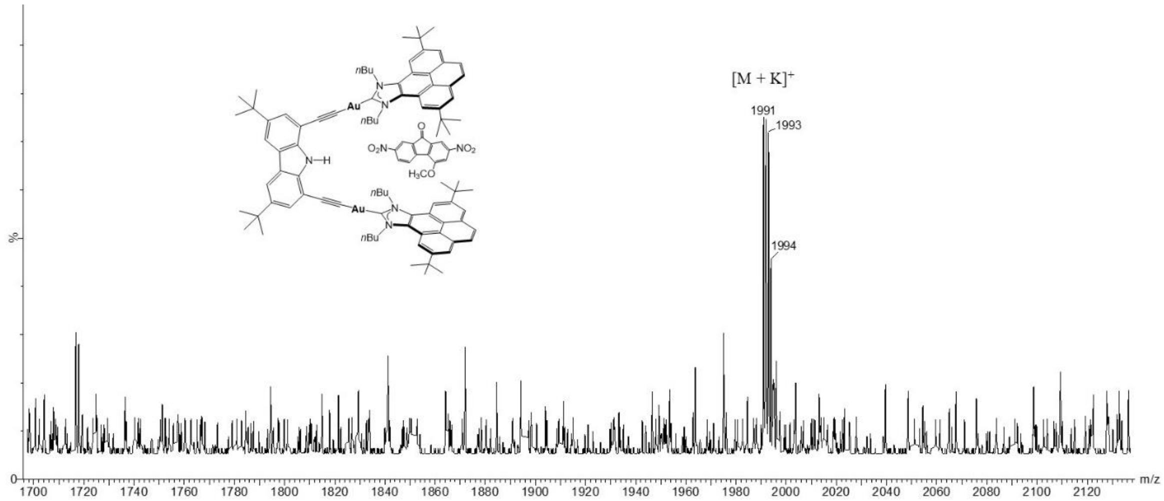

Figure S18. Selected region of the HRMS ESI-TOF-MS (positive mode) spectrum of **6@3**.

7. Cyclic voltammetry studies

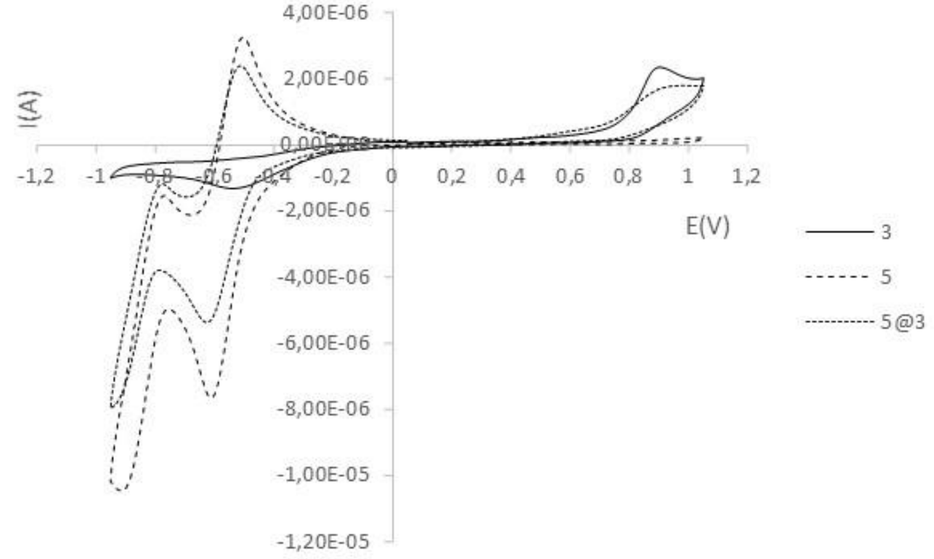

Figure S19. Cyclic voltammetry diagram of **3**, **5** and **5@3**

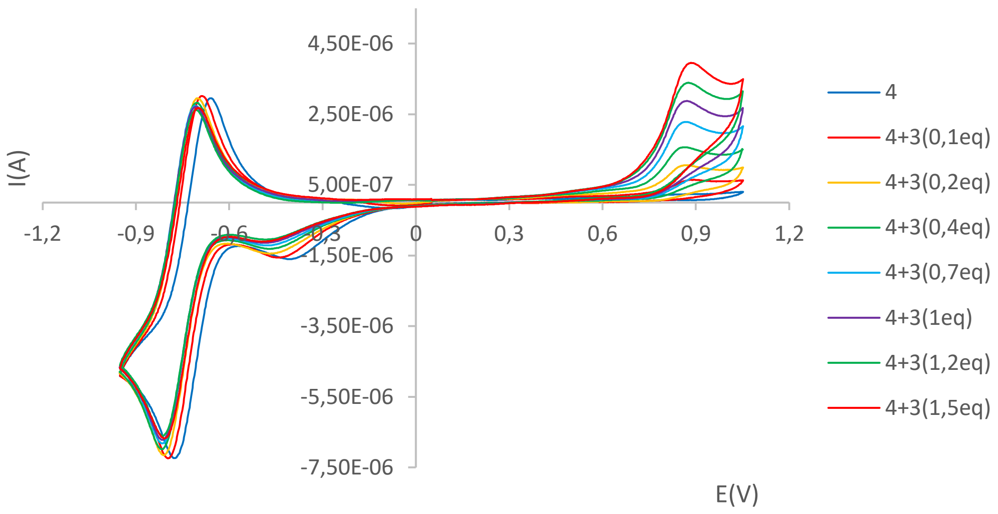

Figure S20. Titration in cyclic voltammetry of **4**(**G**) and **3**(**H**).

8. References

[1] C. Biz, S. Ibañez, M. Poyatos, D. Gusev and E. Peris, *Chemistry-a European Journal* **2017**, *23*, 14439-14444.

[2] Y. Liang, P. Zhang and C. J., *Chem. Sci.* **2013**, *4*, 1330-1337.

[3] K. M. C. Wong, L. L. Hung, W. H. LAm, N. Zhu and V. W. W. Yam, *J. Am. Chem. Soc.* **2007**, *129*, 4350-4365.

[4] N. G. Connelly and W. E. Geiger, *Chemical Reviews* **1996**, *96*, 877-910.

[5] O. V. Dolomanov, L. J. Bourhis, R. J. Gildea, J. A. Howard and H. Puschmann, *Journal of Applied Crystallography* **2009**, *42*, 339-341.

[6] G. M. Scheldrick, *Acta Crytallogr*, *Sect A* **2008**, *64*, 112-122.

[7] G. M. Scheldrick, *Acta Crytallographica a-Foundation and Advances* **2015**, *71*, 3-8.
